# Influence of Zwitterionic Buffer Effects with Thermal Modification Treatments of Wood on Symbiotic Protists in *Reticulitermes grassei* Clément

**DOI:** 10.3390/insects12020139

**Published:** 2021-02-06

**Authors:** Sónia Duarte, Lina Nunes, Davor Kržišnik, Miha Humar, Dennis Jones

**Affiliations:** 1 LEAF (Linking Landscape, Environment, Agriculture and Food) Research Centre, Instituto Superior de Agronomia, Universidade de Lisboa. Tapada da Ajuda, 1349-017 Lisboa, Portugal; 2 LNEC, National Laboratory for Civil Engineering, Structures Department, Av. do Brasil, 101, 1700-066 Lisbon, Portugal; linanunes@lnec.pt; 3 cE3c, Centre for Ecology, Evolution and Environmental Changes/Azorean Biodiversity Group, University of the Azores, 9700–042 Angra do Heroísmo, Portugal; 4 Biotechnical Faculty, University of Ljubljana, SI-1000 Ljubljana, Slovenia; davor.krzisnik@bf.uni-lj.si (D.K.); miha.humar@bf.uni-lj.si (M.H.); 5 Department Wood Science and Engineering, Luleå University of Technology, Forskargatan 1, S-93197 Skellefteå, Sweden; dennis.jones@ltu.se; 6 Department of Wood Processing and Biomaterials, Faculty of Forestry and Wood Sciences, Czech University of Life Sciences Prague, Kamýcká 1176, 16521 Praha 6–Suchdol, Czech Republic

**Keywords:** bicine, symbionts, termite gut, thermal modification, tricine, wood, *Reticulitermes grassei*

## Abstract

**Simple Summary:**

Over the past thirty years, the thermal modification of wood has become a universally recognised and commercialised wood modification process. Thermal modifications may affect wood properties, either positively (dimensional stability and decay resistance) or negatively (mechanical properties). The combination of the impregnation of specific reagents with thermal modification may help to overcome the negative effects on wood properties. In this study, we evaluate the effect of a combination of two zwitterionic buffers, bicine and tricine, and thermal modification of two wood species (beech and spruce) against subterranean termites and their symbiotic fauna. Bicine and tricine treatments alone had a clear influence on wood mass loss and termite survival. The flagellate protist symbiotic community was affected by the treatments and responded differently to them, as a highly adaptable community. However, the combination of bicine with the thermal modification showed a negative effect on termites and their symbionts on both wood species. The combination of these different factors should be further investigated, as these results seem to be promising with regard to the enhancement of the termite resistance of wood.

**Abstract:**

The majority of thermal modification processes are at temperatures greater than 180 °C, resulting in a product with some properties enhanced and some diminished (e.g., mechanical properties). However, the durability of thermally modified wood to termite attack is recognised as low. Recent attempts at combining thermal modification with chemical modification, either prior to or directly after the thermal process, are promising. Buffers, although not influencing the reaction systems, may interact on exposure to certain conditions, potentially acting as promoters of biological changes. In this study, two zwitterionic buffers, bicine and tricine, chosen for their potential to form Maillard-type products with fragmented hemicelluloses/volatiles, were assessed with and without thermal modification for two wood species (spruce and beech), with subsequent evaluation of their effect against subterranean termites (*Reticulitermes grassei* Clément) and their symbiotic protists. The effect of the wood treatments on termites and their symbionts was visible after four weeks, especially for spruce treated with tricine and bicine and heat treatment (bicine HT), and for beech treated with bicine and bicine and heat treatment (bicine HT). The chemical behaviour of these substances should be further investigated when in contact with wood and also after heat treatment. This is the first study evaluating the effect of potential Maillard reactions with zwitterionic buffers on subterranean termite symbiotic fauna.

## 1. Introduction

The subterranean termites are known as major pests of applied wood and their control has always been challenging, and even more so in an environmentally friendly way. There are several methods and approaches for obtaining this control, which often need to be combined to gain a degree of synergy of their effects [1]. For example, heat-treated wood may exhibit different degrees of resistance to termites, according to the wood product and heat treatment technique, among other factors (e.g., [2,3,4]), but is generally recognised as low [5].

The thermal modification of wood is accepted as being environmentally friendly and is recognised as the most commercialised of the wood modification processes to date [6,7]. The majority of thermal treatments of wood involve temperatures greater than 180 °C, resulting in a product with some properties enhanced (e.g., dimensional stability, durability, aesthetical colour) and some diminished (e.g., mechanical properties). At temperatures between high-temperature kiln drying and conventional thermal modification, changes in properties are limited [8]. The loss of mechanical strength has been a limitation to the use of conventional thermally modified wood in certain products. The mechanical strength reduction is linked in varying degrees to the release of acidic volatile species, the acid-catalysed depolymerisation of the hemicelluloses present, and the plasticisation and redistribution of the lignin components present.

More recently, there have been attempts to combine thermal modification with chemical modification methods. The combination of thermal modification at moderate treatment temperatures with wax impregnation has been reported [9] for the tropical wood *Pterocarpus macrocarpus* Kurz. The reduction in the modulus of rupture (MOR) resulting from mild thermal treatment at 150 °C was eliminated as a result of wax impregnation. This continued the work outlined by Humar and collaborators [10], where Montan wax was impregnated into Norway spruce (*Picea abies* (L.) H. Karst.) prior to thermal modification at temperatures between 185 and 230 °C. The use of resins has also been reported, such as the treatment of eucalyptus with melamine–urea–formaldehyde resin prior to heat treatment [11] and the impregnation of beech (*Fagus sylvatica* L.), ash (*Fraxinus excelsior* L.), lime (*Tilia* spp.) and poplar (*Poplar* spp.) with methylated melamine formaldehyde resin (MMF) after thermal treatment at 230 °C [12]. The potential of the impregnation of compounds such as vinylic-polyglycerol, vinylic-glycerol, and maleic anhydride followed by thermal modification has been assessed as a means of achieving a non-biocidal antifungal and anti-termite wood treatment [13].

The darkening of the wood colour during thermal modification has been suggested as a result of a Maillard reaction [14,15]. More commonly associated within food science, this reaction is the result of an amine reacting with a reducing sugar induced by heat. The possibility of a Maillard reaction occurring in wood modification was proposed by Hauptmann and collaborators [16] for the reaction of xylose with tricine. The Maillard reaction may increase the wood dimensional stability, through the bulking of the cell wall, depending on the chemical components used to perform the reaction [17]. Tricine was shown to react with hemicellulose, resulting in an increase in wood hardness [16]. Additional examination of the potential of tricine and the tertiary amine compound bicine [2-(Bis(2-hydroxyethyl) amino) acetic acid] as potential wood modification agents was recently undertaken [18]. Evaluation of treated samples by Fourier-transform infra-red spectrometry [19] indicated the reagents underwent reaction with wood after impregnation into samples, followed by thermal modification at 160 °C, supported by hierarchical analyses of the spectral data.

Zwitterionic buffers are neutral molecules with a positive and a negative electrical charge, with some advantages regarding other types of buffers, due to their stability and reduced membrane permeability [20,21]. The advantages of studying these substances for wood treatment is their low human and environmental toxicity, low price, and relative ease of preparation, as described in the scientific literature (e.g., [20]). Bicine and tricine, both considered within this study, are examples of zwitterionic buffers; these substances are commonly used as non-fermentable buffering agents for some reactions, including the manufacture of primary cells (bicine) and animal tissue culture (tricine) [22,23].

The pH range of bicine is 7.6–9.0, and that of tricine is 7.4–8.8 [23]. However, these values may decrease with a temperature increase, though this does not interfere significantly with their ionic strengths (for a temperature range between 25 and 50 °C [22]).

Tricine has been described as an efficient scavenger of -OH radicals [24], though later research suggested lower activity of bicine and tricine to hydroxyl radicals compared to other Good’s buffers [25]. 

Buffers are designed to have little influence the reactions in which they are used, so it is important to investigate the compatibility of the buffer with the reaction system not only regarding the buffering range but also with regard to other possible buffer interactions. Such examples of this may be the possible impacts of the buffer on cell growth and survival, the interactions of buffers with macromolecules (for example, tricine may form complexes with DNA, and zwitterionic buffers influence mRNA expression of some reaction systems), and the impact of buffers on redox studies [21]. Buffers, although selected so as not to influence the reaction systems, may have some interactions when exposed to some conditions and act as promoters of biological changes on those systems.

Tricine has been proposed to form a xylosamine at temperatures around 103 °C (as typical of laboratory drying conditions) [13]. Further rearrangements were suggested, whereby the xylosamine was converted to a ketosamine. Hauptmann and collaborators [16] represented one of the first documented studies into the Maillard reaction in wood, even though the concept has long been considered a probability. By better understanding the depolymerisation/degradation process within the hemicellulose, it may be possible to apply Maillard-type reactions and react the sugar moieties within the cell wall matrix, instead of being volatilised.

The Maillard reaction is dependent upon the interaction between the amine and the sugar, with the ease of reaction typically being greater for primary amines compared to secondary amines (representing the reactivity of the N-H bond). Typically, tertiary amines are not expected to react in a similar way, since there are no N-H bonds present. However, for compounds such as bicine, there may be an opportunity for thermal degradation reactivating the nitrogen group present. On the other hand, it is known that N, N-bis(2-hydroxyethyl) glycine (bicine) and N-(2-hydroxy-1,1-bis(hydroxymethyl) ethyl) glycine (tricine) are important hydrogen ion buffers for biological media, and their use has been noted in studies into the buffering of termite gut protists (e.g., [21]). The advantage of the application of Maillard reaction to wood relies on its simplicity, as it involves an aqueous process, and it is ignited by heat [17].

The bulking of cell wall and the wood hardening process, referred to as effects of the Maillard reaction, may contribute positively to an increase in treated wood termite resistance, as these properties difficult the task of attacking and processing wood.

Moreover, the lower termites’ hindgut, which harbours their symbiotic fauna (prokaryotes and flagellate protists), is naturally designed to provide a rich and complex environment able to fulfil the specific requirements, regarding pH, oxygen, and hydrogen, of the several different species of symbionts living inside termites [26]. Therefore, various gradients of pH, oxygen and hydrogen exist, fuelled by a combination of host and symbiont activities inside the termite hindgut [27]. The bulk of the cellulose digestion process is performed by flagellated protists, which belong to two separate lineages of the unicellular eukaryotes: the order Oxymonadida (Phylum Preaxostyla) and the phylum Parabasalia [28,29,30]. There is growing interest in the lignocellulolytic degradation process performed by the termite and symbiotic fauna holobiont [31]. Flagellate protists seem to be sensitive to the termite diet, which may lead to changes in the flagellate protist species composition and community structure [32,33,34]. 

The analysis of termite survival and consumption of wood (either by measuring wood mass loss and/or grading the attack) are common methods to evaluate wood treatments’ efficacy against termites [35]. The effect of these wood treatments on symbiotic flagellate protist community may also shed a light on the treatment mode of action and the extent of effects on termites. The advantages of this mode of action, focused on the effects on the termite symbiotic flagellate protist community, will be the specificity of the treatments, which mean that it will probably have fewer side effects on non-target organisms.

This work objectives are to evaluate the effect of zwitterionic buffers and heat treatment on 1) subterranean termite health and 2) the subterranean termite symbiotic flagellate protist community.

## 2. Materials and Methods

### 2.1. Wood Treatment

Wood samples of spruce (*P. abies*) and beech (*Fagus sylvatica* L.) of dimensions 30 × 10 × 10 mm (LxRxT, Gorenjska, Slovenia) were treated with solutions of tricine and bicine (obtained from Fisher Scientific, Hampton, NH, USA), using a vacuum impregnation method: the samples were placed in containers and weighed down, 1 M solutions of bicine or tricine in distilled water were respectively added; the impregnation schedule followed the full cell process, 20 min of vacuum of 10 kPa followed by two hours of overpressure at 900 kPa and at the end for 10 min of vacuum of 10 kPa. Before and after the impregnation, the samples were dried and weighed to determine uptake. The treated samples were allowed to dry under low heating conditions (50 °C).

Once dry conditions had been achieved, samples were then thermally treated using a modified process to the one described by Rep and collaborators [36], whereby a reduced maximum temperature of 160 °C was used (instead of the conventional 180–230 °C employed for thermal modification of wood) under vacuum. This was to minimise the thermal degradation of the bicine and tricine respectively at temperatures around 180 °C. Spruce and beech samples were also subject to the same heat treatment process of 160 °C without any impregnation.

The following combinations of treatments were evaluated against subterranean termites and their protist symbionts for both wood species: (1) untreated; (2) heat-treated (HT); (3) bicine; (4) bicine and heat treatment (bicine HT); (5) tricine; (6) tricine and heat treatment (tricine HT).

### 2.2. Effects on Subterranean Termites

Subterranean termites belonging to the species *Reticulitermes grassei* Clément, and to three different colonies, were captured in a pine forest (*Pinus pinaster* Aiton), in Sesimbra, Setúbal district of Portugal. Each colony was captured on a dead wood log (approximately 1 m long and 0.08 m diameter) more than 100 m apart. Logs were brought to the laboratory and kept in a conditioned room at 24 ± 1 °C and 80% ± 5% relative humidity. Groups of 150 termite workers were captured manually and established in 200 mL glass jars with moistened sand (Fontainebleau sand and water; 4:1 *v*/*v*) as substrate. Previously treated spruce and beech wood (as explained in the previous point) was offered to the termites as food, and also pine controls were established. Three replicates per treatment were then placed in contact with the termites and the test was run for four weeks in the described conditions. Maritime pine test specimens with the same dimensions were also included as internal virulence controls [37,38].

The initial moisture content of the blocks was measured in sets of three additional replicates per treatment and these values were used to determine the theoretical initial dry mass (IDM) of the exposed specimens. At the end of the trial, the final moisture content was recorded, and the mass loss was obtained according to Equation (1).

% mass loss = (FDM − IDM)/IDM × 100(1)

where FDM is the dry mass of the block at the end of the test. The survival rate (%) of the termites was also recorded. All wood blocks were graded according to termite attack using the scale: 0 = no damage; 1 = attempted attack; 2 = slight damage; 3 = superficial and inner damage; 4 = heavy inner damage [37].

### 2.3. Scanning Electron Microscopy (SEM)

Termites of one of the colonies used in 2.2 were used for microscopy. Hindguts of 50 workers were distributed on 250 μL of Trager U medium [39], cut open and slightly homogenised to release their contents. The contents were put on a 0.1% poly-L-lysine coated surface, and then were fixed in 1:1 v/v glutaraldehyde 2.5%/formaldehyde 2.5% overnight. After centrifugation, and three buffer washes, of 15 min each, samples were post-fixed in 1% osmium tetroxide for 30 min. The samples were dehydrated using a graded ethanol series, 15 min in each concentration: 30%, 50%, 70%, 80%, 90%, and three times in 100% ethanol, and then put onto the drier agent hexamethyldisilazane. Dried samples were mounted on stubs and sputter coated with gold before observing with a scanning electron microscope JEOL JSM-5200LV (JEOL, Croissy-Sur-Seine, France) at Faculty of Sciences of Lisbon University.

### 2.4. Effects on Symbiotic Protists

The termites used belonged to the same colonies referred on Section 2.2. Termite workers in the 4th instar of development were selected. Groups of 50 termite workers of each colony, randomly chosen, were exposed to the different wood samples, and five termites per type of wood treatment were evaluated for their symbiotic flagellate protist community diversity and abundance after: 24 h, one week, two weeks, three weeks, and four weeks (end of the trials). Flagellate protist diversity and abundance were evaluated by direct observation under a microscope. The quantification of flagellate protists was performed with a haemocytometer, in accordance with a previously described method [40]. The three different colonies’ symbiotic fauna was compared using termites feeding on the original wood where they were captured.

Flagellate protists were distinguished and identified according to species or major taxa descriptions [41,42,43,44], based on morphological characteristics, separated into nine morphotypes, and quantified.

### 2.5. Statistical Analysis

In order to detect significant (*p* < 0.05) variation in termite survival and wood mass loss caused by different wood treatments, the data obtained were submitted to an ANOVA, and when the result was considered to be significant (for *p* < 0.05), Tukey’s honestly significant difference test (HSD) was done. The grade of termite attack was also analysed with a non-parametric Kruskal–Wallis analysis (and a post hoc pairwise Wilcoxon test), as well as data on symbiotic flagellate protists, as the assumptions of data normality were not respected. All these analyses were performed with RStudio (RStudio Team 2015) v 0.99.467 and R-3.1.2 (R Foundation for Statistical Computing, Vienna, Austria).

## 3. Results

### 3.1. Efficacy against Subterranean Termites

Regarding beech, no significant difference was identified in termite survival (F = 1.83; dF = 5; *p* = 0.181), despite the lower values of termite survival for termites fed with bicine and bicine and heat treatment (bicine HT) beech (Table 1). This lack of statistical significance may be explained by strong variation within the results of different trials belonging to the same category. However, the results for wood mass loss (F = 14.40; dF = 5; *p* < 0.001) and termite attack grade (Table 2) corroborate the differences observed in termite survival rate, and two groups are visible: 1) untreated beech and heat-treated beech (HT), and 2) bicine and bicine and heat treatment (bicine HT), also including tricine, which had significantly lower mass loss compared to group 1.

Regarding termite survival rate, the results show a clear difference (F = 6.28; dF = 5; *p* = 0.004) between spruce submitted to 1) untreated spruce and heat treated spruce and 2) bicine and heat treatment and tricine, which caused significantly lower termite survival rate. For mass loss results, the differences are significant (F = 26.96; dF = 5; *p* < 0.001) between 1) untreated and HT spruce and 2) bicine and bicine HT spruce, with the latter having lower mass losses. Termite attack grade showed that bicine HT and tricine had lower attack grading, although no significant differences among them were detected (spruce: χ2 = 10.50; df = 5; *p* = 0.062; beech: χ2 = 10.88; df = 5; *p* = 0.054).

### 3.2. Effects on Symbiotic Protists

The three colonies exhibited no significant differences regarding their symbiotic protists (χ2 = 0.73; df = 2; *p* = 0.692). Nine morphotypes were found and identified based on morphological characteristics (Table 3). Variations in protist diversity and abundance were evaluated for all treatments tested (Table 4-spruce; Table 5-beech).

Some morphotypes were shown to be susceptible to some treatments. For example, n1 (*Trichonympha* sp.) was susceptible to all treatments for both wood species after 28 days of exposure. All wood treatments showed a clear negative impact over time on termites, regarding this morphotype. For spruce, tricine (*p* = 0.004) and tricine HT (0.042) exert negative influences by day 1 (χ2 = 20.58; df = 6; *p* = 0.002), while bicine (*p* = 0.012) and bicine HT (*p* = 0.031) exert negative influences by day 7 (χ2 = 25.26; df = 6; *p* < 0.001), and heat-treated spruce-fed termites (*p* = 0.003) exhibited a lower number of n1 protists by day 14 (χ2 = 51.25; df = 6; *p* < 0.001). For beech, the negative effects of different wood treatments, relatively to initial pine fed termites was visible on day 7 (χ2 = 21.29; df = 6; *p* = 0.002) for tricine (*p* = 0.029) and tricine HT (*p* = 0.011), which was also different from termites fed on spruce controls (*p* = 0.026), although later tricine-treated beech-fed termites showed a recovery in these protists’ abundance. For bicine (*p* = 0.018) and bicine HT (*p* = 0.012), effects were visible after day 14 (χ2 = 25.01; df = 6; *p* < 0.001) relative to pine, and heat-treated beech (*p* < 0.001, *p* = 0.033, respectively) showed a stronger negative effect on *Trichonympha* sp. abundance on day 28 (χ2 = 95.30; df = 6; *p* < 0.001).

**Figure 1:**
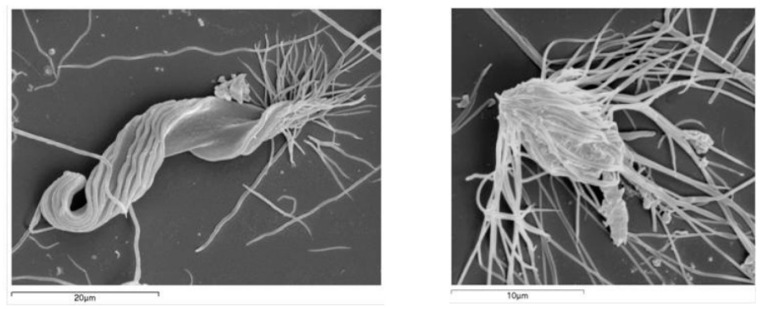
Scanning electron micrograph for: morphotype n2 (*Pyrsonympha* sp.; Preaxostyla, Oxymonadida) (**left**), and morphotype n8 (*Microjoenia hexamitoides*; Parabasalia, Spirotrichonymphida) (**right**).

The abundances of morphotype n2 (*Pyrsonympha* sp1; Figure 1) fluctuated, but were generally significantly lower than pine control fed termites, for all treatments and both wood species (spruce: χ2 = 75.97; df = 6; *p* < 0.001; beech: χ2 = 25.26; df = 6; *p* < 0.001). For spruce, the negative effect on this morphotype abundance was observed at the end of the trials (day 28) with wood treated with bicine- (*p* = 0.023) and bicine HT-fed termites (*p* < 0.001), which exhibited a lower abundance than untreated spruce fed termites. For beech, this effect was stronger at the end of the trials on termites fed with tricine (*p* = 0.008) and bicine HT (*p* = 0.004) wood relative to termites fed with untreated beech.

The abundance of *Dinenympha gracilis* Leidy, corresponding to morphotype 3, decreased with all treatments, except for heat-treated beech, until day 14, then a recovery was observed on day 14 and afterwards a new decrease was observed until the end of the trials (χ2 = 27.30; df = 6; *p* < 0.001). For spruce, the negative effect of the treatments involving zwitterionic buffers was also observed, comparatively to pine, untreated spruce, and heat-treated spruce (χ2 = 57.37; df = 6; *p* < 0.001). *Holomastigotes elongatum* Grassi, corresponding to morphotype n4, decreased its abundance throughout the trials for both wood species, including treatments and controls. This protist disappeared on bicine HT spruce (after 14 days) and tricine HT spruce (after 21 days of exposure), and beech treated with bicine and tricine (both after 21 days of exposure).

For both the control and heat-treated spruce, the abundance of *Dinenympha fimbriata* Kirby (morphotype n5) decreased; for the other treatments, they disappeared after 7 (bicine, bicine HT and tricine HT spruce) or 14 (tricine-treated spruce) days of exposure. For beech, this protist disappeared after 7 (control, bicine, and tricine HT beech), 14 (tricine, bicine HT beech), or 21 (heat-treated) days of exposure. The wood treatments did not seem to negatively affect morphotype n6 (belonging to Hypotrichomonadidae family), except for spruce treated with bicine, as the abundance fluctuated over time; however this protist’s density was generally low and no significant effects were detected. Morphotype n7 (*Pyrsonympha* sp.2) showed fluctuations on abundance, although no significant effects were detected.

Relative to morphotype n8 (putatively *Microjoenia hexamitoides* Grassi; Figure 1), for spruce, a significant decrease in its abundance was detected on day 1 (χ2 = 26.58; df = 6; *p* < 0.001), relative to the pine controls, regarding untreated spruce (*p* < 0.001), tricine (*p* = 0.007), and tricine HT (*p* = 0.046). On day 7, an increase in this protist’s abundance on untreated and heat-treated spruce-fed termites was observed, and on the contrary, for all the remaining treatments, a significant decrease (χ2 = 37.46; df = 6; *p* < 0.001) was observed. From day 21 onwards, untreated and heat-treated spruce demonstrated abundances similar to pine, while all the remaining treatments showed significantly lower abundances of *M. hexamitoides* (χ2 = 29.29; df = 6; *p* < 0.001). For beech, a significant decrease in this protist on termites fed with beech treated or untreated woods was observed, except for heat-treated beech. From day 21, all treatments showed termites with significantly lower *M. hexamitoides* abundances comparatively to pine-fed termites (χ2 = 45.20; df = 6; *p* < 0.001). On day 28, regarding beech controls, bicine (*p* = 0.006), bicine HT (*p* < 0.001) and tricine (*p* < 0.001) showed termites with significantly lower *M. hexamitoides* abundances (χ2 = 95.30; df = 6; *p* < 0.001).

*Spirotrichonympha flagellata* Grassi (morphotype n9) abundances on days: 14 (χ2 = 34.89; df = 6; *p* < 0.001), 21 (χ2 = 66.75; df = 6; *p* < 0.001) and 28 (χ2 = 45.36; df = 6; *p* < 0.001) on termites fed on the spruce treated with bicine and tricine were significantly lower than on termites fed on pine. Bicine-treated spruce resulted in lower abundances relative to all the remaining spruce untreated or treated wood in the end of the trials. For beech, the results show a clear tendency towards a decrease in this morphotype’s abundance in termites fed with wood treated with the zwitterionic buffers, especially on days 7 and 14 (χ2 = 25.18; df = 6; *p* < 0.001; χ2 = 41.67; df = 6; *p* < 0.001, respectively). On day 28, untreated and heat-treated beech and pine fed termites showed termites with significantly higher *S. flagellata* abundances relative to the remaining treatments (χ2 = 92.38; df = 6; *p* < 0.001).

Lower termite survival rates were observed for bicine HT spruce and tricine treated spruce. A significant decrease in the abundances of most protists was observed, comparing to pine fed termites (except for morphotypes n4, n6 and n7). Bicine HT spruce-fed termites showed a significant decrease in *Trichonympha* sp., *Pyrsonympha* sp.1, *D. gracilis*, and *H. elongatum*, while tricine-treated spruce-fed termites showed significantly lower abundances of *D. gracilis* and *M. hexamitoides*, both relative to untreated spruce-fed termites.

For beech, lower survival rates were observed for wood treated with bicine and bicine HT. Bicine-treated wood-fed termites showed significantly lower abundances of *Trichonympha* sp., *Pyrsonympha* sp.1, *D. gracilis*, *M. hexamitoides*, and *S. flagellata*, relative to pine-fed termites, and protists *Pyrsonympha* sp.1, *D. gracilis*, *M. hexamitoides*, and *S. flagellata* relative to untreated beech-fed termites. Bicine HT beech-fed termites showed significantly lower abundances of morphotypes *Trichonympha* sp., *Pyrsonympha* sp.1, *M. hexamitoides* and *S. flagellata*, relative to pine-fed termites, and morphotypes *Pyrsonympha* sp.1, *D. gracilis*, *H. elongatum*, *M. hexamitoides*, and *S. flagellata* relative to untreated beech fed termites. So, the most abundant protists decreased their abundances.

## 4. Discussion

Both tricine and bicine were found to have an effect on termite survival. The lower survival rates observed in the trials involving the evaluation of termite survival and wood mass loss may be related with decreases in the numbers of flagellate protists, both for spruce and beech. Three morphotypes seem to have a major role in this, as their decrease is linked to lower termite survival rates and lower mass losses (for spruce-treatment with bicine HT and with tricine; for beech-wood treated with bicine and bicine HT): *Pyrsonympha* sp.1, *M. hexamitoides* and *S. flagellata*, respectively. These morphotypes are the most abundant, and therefore probably most important species/group of species of protists living inside *R. grassei* hindgut [40].

*Trichonympha* sp. is one of the biggest and most motile flagellate protists, searching actively for food and moving quickly and easily through the hindgut environment; therefore, the recovery observed after 21 days of exposition to untreated and tricine treated spruce wood could be attributed to possible cannibalism or necrophagy of this protist on living or dead protists belonging to the same or to other species [42]. Another hypothesis is the trophallaxis between termites, which contributes to symbiotic refaunation.

Low densities of some morphotypes (*H. elongatum*, morphotype n6 hypotrichomonad) may impair the discussion of those results; however, for *H. elongatum*, heat treatment seems to be negative. Heat treatment possibly causes alterations in the wood that affect this protist negatively. *Dinenympha fimbriata* seemed to be negatively affected by all treatments and also controls at the end of the trials, and so it is hypothesised that a laboratory environment may negatively influence this morphotype’s survival and abundance inside the subterranean termite hindguts. The laboratory environment may influence termites due to the less varied diet, the scattering of social interactions, and the lack of contact with soil and other components of their natural environment [45]. Despite this, the survival of the termites did not seem to be directly affected by the absence of these protists.

The termite symbiotic flagellate protist community seems to be organised into a core group (including *Pyrsonympha* sp.1, *M. hexamitoides*, and *S. flagellata*, three of the most abundant protists living inside *R. grassei* [40]), which is crucial for termite survival, and a flexible secondary symbiont community (including, for example, *D. fimbriata*, morphotype n6 hypotrichomonad or *Pyrsonympha* sp.2), all contributing with different loads to host fitness [40]. However, severe changes on symbiotic flagellate protist communities may lead to termite mortality, regardless of the possible redundancy of some protist species.

The thermal treatment of wood may lead to a variable degree of decomposition of wood components according to the type of heat treatment applied [46]. The common side effects of thermal treatment of wood may include: loss of functional groups (carboxyl, hydroxyl, and acetyl), increase in carbon content, solubilisation of lignin, pH decrease and decomposition of sugars [46]. All these effects interfere directly (decrease in possible sources of energy available) and indirectly (alter the chemical stability of the termite hindgut) with the lignocellulose digestion process [26]. Both hypotheses will exert effects on the termite symbiotic fauna. For example, *S. flagellata* seemed to be positively associated with spruce and beech heat treatment, although when bicine and tricine treatments were together with heat treatment, this effect was not visible. Consequently, another mode of action may be triggered by the chemical and physical changes caused in the termite hindgut through the ingestion of wood treated with zwitterionic buffers and/or heat treatment. As the maintenance of an anoxic environment in some parts of the termite hindgut is one of the tasks of the bacterial symbionts of termites, any alteration of the physical-chemical equilibrium of the hindgut will surely affect all of the symbiotic community, both bacteria and flagellate protists. Bacterial symbionts may be free living or symbiotic to flagellate protists (either endo- and ectosymbionts) and are closely related and have a direct influence on flagellate protist survival (e.g., [47]). Additionally, the maintenance of an anoxic environment in some parts of the termite hindgut is one of the tasks of the bacterial symbionts of termites, and an alteration of this community may cause severe changes

The changes in abundances and diversity of the flagellate protist community of the subterranean termites tested may involve a possible result of termite workers’ adaptation to the laboratory conditions and new diet, already observed in previous studies [48]. These changes prove their adaptation ability to new conditions, although in some cases, termite mortality is not avoided, despite this adaptation effort.

The Maillard reaction has been investigated for wood treatment, mainly for the enhancement of the fungal decay resistance and increase dimensional stability [17]. However, to date, bicine and tricine have not been traditionally used for wood treatment and enhancement, and specifically the termite resistance of treated wood has not been evaluated. The effect of the wood treatments on termites and their flagellates was visible after four weeks of exposure, especially for spruce treated with tricine and bicine HT treatment, and for beech treated with bicine and bicine HT. The chemical behaviour of these substances should be further investigated when in contact with wood and also after heat treatment.

The zwitterionic buffers used have a high pH range (bicine: 7.6–9.0; tricine: 7.4–8.8) when comparing with the hindgut pH of lower termites (6–6.5, for *Reticulitermes lucifugus* (Rossi) paunch [49]). The termite hindgut is a highly structured environment, with micro niches with gradients pH, oxygen and hydrogen and any alteration on the pH may lead to the disruption of the hindgut chemical stability [26], probably originating changes in the termite symbiotic community, either bacteria or flagellate protists, as their survival is closely related.

However, further investigation on the behaviour of the zwitterionic buffers when impregnated into wood (both softwood and hardwood) after heat treatment and also their direct effect on termites is needed. Another hypothesis for the chemical stability of hindgut disruption is the reaction of these buffers with substances existing inside the termite hindgut, as they are described as ion scavengers [24].

## 5. Conclusions

Bicine and tricine treatments alone had an influence on the survival of the termites. The trials showed the plasticity of termite hindgut flagellate protist community to the exposure to different wood treatments, involving two zwitterionic buffers and the heat treatment of two different wood species.

The characterisation of the chemical behaviour of both of these substances inside wood and after heat treatment will allow the understanding of the mode of action towards the symbiotic fauna of the subterranean termites, as the results obtained in this study seem promising.

## Figures and Tables

**Table 1:** Average results of termite survival rates, wood mass loss (%; and standard deviation) for different wood species (pine, spruce and beech) and the treatments: untreated, heat-treated (HT), bicine, bicine and heat treatment (bicine HT), tricine, tricine and heat treatment (tricine HT), after four weeks of exposure. Values followed by different letters within the same column were considered to be significantly different (*p* < 0.05; Tukey´s honestly significant difference test). The comparisons were made for different treatments within the same wood species.

Wood Species	Treatment	Survival (%)		Mass Loss (%)	
Pine	Untreated	84.4 ± 7.4	-	18.3 ± 2.9	-
Spruce	Untreated	88.4 ± 6.3	a	19.5 ± 0.8	a
	HT	90.8 ± 4.5	a	19.0 ± 1.9	a
	Bicine	64.2 ± 38.9	ab	11.7 ± 1.0	b
	Bicine HT	21.1 ± 18.7	b	10.8 ± 1.5	b
	Tricine	11.3 ± 17.4	b	11.5 ± 1.1	ab
	Tricine HT	62.3 ± 31.2	ab	17.5 ± 0.8	ab
Beech	Untreated	82.0 ± 9.7	a	15.9 ± 0.8	a
	HT	87.2 ± 2.7	a	14.8 ± 0.8	a
	Bicine	38.0 ± 22.0	a	12.3 ± 0.3	b
	Bicine HT	57.6 ± 47.0	a	11.4 ± 1.3	b
	Tricine	71.4 ± 17.2	a	14.2 ± 0.3	b
	Tricine HT	79.9 ± 16.8	a	13.0 ± 0.0	ab

**Table 2:** Grade of termite attack for different wood species (pine, spruce, and beech) and the treatments: untreated, heat treated (HT), bicine, bicine and heat treatment (Bicine HT), tricine, tricine and heat treatment (Tricine HT), after four weeks of exposure, for each replicate. The wood blocks were graded using the scale: 0 = no damage; 1 = attempted attack; 2 = slight damage; 3 = superficial and inner damage; 4 = heavy inner damage [37].

Wood Species	Treatment					
	Untreated	HT	Bicine	Bicine HT	Tricine	Tricine HT
Spruce	4	4	3	4	4	4
	4	4	3	3	3	4
	4	4	3	1	2	4
Beech	4	4	3	4	4	4
	4	4	3	3	4	4
	4	4	3	2	3	4
Pine	4	-	-	-	-	-
	4	-	-	-	-	-
	4	-	-	-	-	-

**Table 3:** Flagellate protist identification to morphotypes (n1 to n9) based on morphological characters.

	Phylum	Class	Order	Family	Genus	Species
n1	Parabasalia	Trychonymphea	Trichonymphida	Trichonymphidae	*Trichonympha*	
n9		Spirotrichonymphea	Spirotrichonymphida	Holomastigotoididae	*Spirotrichonympha*	*S. flagellata*
n4					*Holomastigotes*	*H. elongatum*
n8					*Microjoenia*	*M. hexamitoides*
n6		Hypotrichomonadea	Hypotrichomonadida	Hypotrichomonadidae		
n2	Preaxostyla		Oxymonadida		*Pyrsonympha* sp.1	
n7					*Pyrsonympha* sp.2	
n3					*Dinenympha*	*D. gracilis*
n5						*D. fimbriata*

**Table 4:** Average number (and standard deviation) of flagellate protists belonging to different morphotypes (n1 to n9) after 1, 7, 14, 21, and 28 days of exposure; termites were exposed to untreated pine (Pine), untreated spruce (Control), heat-treated spruce (HT), spruce treated with: bicine (B), bicine and heat treatment (BHT), tricine (T), tricine and heat treatment (THT). Different capital letters within the same column indicate significant differences (*p* < 0.05) between pine and the rest of the treatments for each day of observation; different small letters within the same column indicate significant differences (*p* < 0.05) among spruce treatments for each day of observation.

	Treatment	n1	n2	n3	n4	n5	n6	n7	n8	n9
	Pine	0.86 ± 0.78 A	12.06 ± 3.28 A	2.62 ± 1.84 A	0.24 ± 0.48 A	0.30 ± 0.65 A	0.06 ± 0.24 A	0.22 ± 0.58 A	4.56 ± 2.84 A	1.42 ± 1.05 A
Day 1	Control	0.64 ± 0.76 Aab	8.24 ± 3.53 Ba	2.16 ± 1.34 Aa	0.08 ± 0.28 Aa	0.24 ± 0.60 Aa	0.20 ± 0.41 Aa	0.20 ± 0.41 Aa	1.88 ± 1.05 Ba	1.28 ± 1.21 Aab
	HT	0.76 ± 0.66 Ab	10.84 ± 4.04 Aa	3.92 ± 1.53 Bb	0.32 ± 0.48 Aa	0.44 ± 0.58 Aa	0.04 ± 0.20 Aa	0.28 ± 0.46 Aa	2.92 ± 1.68 Aa	1.52 ± 1.19 Ab
	B	0.44 ± 0.58 Aab	9.40 ± 3.86 Ba	2.36 ± 1.47 Aac	0.28 ± 0.46 Aa	0.16 ± 0.37 Aa	0.12 ± 0.33 Aa	0.08 ± 0.28 Aa	3.40 ± 2.45 Aa	0.88 ± 0.97 Aab
	BHT	0.64 ± 0.81 Aab	9.68 ± 3.58 Ba	2.92 ± 1.53 Aab	0.28 ± 0.46 Aa	0.04 ± 0.20 Aa	0.00 ± 0.00 Aa	0.08 ± 0.28 Aa	3.12 ± 1.90 Aa	0.68 ± 0.69 Ba
	T	0.20 ± 0.41 Ba	9.76 ± 4.56 Aa	1.72 ± 1.14 Aa	0.12 ± 0.33 Aa	0.20 ± 0.41 Aa	0.08 ± 0.28 Aa	0.20 ± 0.41 Aa	2.40 ± 1.29 Ba	0.64 ± 0.76 Ba
	THT	0.36 ± 0.57 Bab	10.32 ± 3.16 Aa	3.56 ± 1.87 Abc	0.24 ± 0.44 Aa	0.20 ± 0.41 Aa	0.12 ± 0.33 Aa	0.08 ± 0.28 Aa	2.80 ± 1.80 Ba	1.24 ± 1.05 Aab
Day 7	Control	0.60 ± 0.76 Aab	8.00 ± 3.35 Ba	1.85 ± 1.19 Aa	0.15 ± 0.37 Aa	0.10 ± 0.28 Aa	0.10 ± 0.33 Aa	0.25 ± 0.41 Aa	2.90 ± 2.27 Ba	1.30 ± 1.19 Aa
	HT	0.72 ± 0.68 Ab	8.92 ± 2.43 Ba	4.20 ± 2.18 Bb	0.12 ± 0.33 Aa	0.12 ± 0.33 Aa	0.12 ± 0.33 Aa	0.12 ± 0.33 Aa	4.60 ± 3.44 Ba	1.32 ± 1.07 Aa
	B	0.28 ± 0.54 Bab	7.68 ± 2.70 Ba	1.24 ± 1.13 Bac	0.20 ± 0.41 Aa	0.00 ± 0.00 Ba	0.12 ± 0.33 Aa	0.04 ± 0.20 Aa	2.64 ± 1.91 Ba	0.56 ± 0.77 Bb
	BHT	0.36 ± 0.57 Bab	7.48 ± 3.92 Ba	0.96 ± 0.89 Bc	0.08 ± 0.28 Aa	0.00 ± 0.00 Ba	0.24 ± 0.44 Aa	0.12 ± 0.33 Aa	1.44 ± 1.56 Ba	0.36 ± 0.57 Bab
	T	0.20 ± 0.41 Ba	7.32 ± 3.20 Ba	2.32 ± 1.25 Aa	0.08 ± 0.28 Aa	0.04 ± 0.20 Aa	0.20 ± 0.41 Aa	0.04 ± 0.20 Aa	2.88 ± 2.49 Aa	0.84 ± 0.80 Bab
	THT	0.44 ± 0.58 Aab	8.64 ± 3.65 Ba	2.16 ± 1.46 Aa	0.08 ± 0.28 Aa	0.00 ± 0.00 Ba	0.04 ± 0.20 Ba	0.08 ± 0.28 Aa	3.12 ± 2.01 Aa	0.72 ± 0.94 Bb
Day 14	Control	0.92 ± 0.76 Aa	9.88 ± 3.31 Bac	4.20 ± 1.91 Ba	0.32 ± 0.56 Aa	0.08 ± 0.28 Aa	0.24 ± 0.52 Aa	0.32 ± 0.56 Aa	3.40 ± 1.80 Aa	1.84 ± 1.18 Aa
	HT	0.28 ± 0.46 Bb	10.2 ± 2.36 Bac	3.36 ± 1.58 Aa	0.08 ± 0.28 Aa	0.04 ± 0.20 Aa	0.12 ± 0.33 Aa	0.28 ± 0.54 Aa	6.20 ± 3.44 Ab	1.20 ± 1.12 Aab
	B	0.24 ± 0.44 Bb	6.32 ± 3.67 Bbc	2.12 ± 1.62 Ab	0.12 ± 0.33 Aa	0.00 ± 0.00 Ba	0.12 ± 0.33 Aa	0.00 ± 0.00 Aa	3.00 ± 2.45 Aa	0.52 ± 0.59 Bb
	BHT	0.08 ± 0.28 Bb	7.84 ± 3.59 Bc	2.08 ± 1.53 Ab	0.00 ± 0.00 Aa	0.00 ± 0.00 Ba	0.08 ± 0.28 Aa	0.12 ± 0.33 Aa	2.84 ± 2.37 Ba	0.76 ± 0.88 Bb
	T	0.16 ± 0.37 Bb	11.48 ± 3.72 Aa	2.24 ± 1.13 Ab	0.12 ± 0.33 Aa	0.00 ± 0.00 Ba	0.16 ± 0.47 Aa	0.08 ± 0.28 Aa	3.44 ± 2.81 Aa	0.64 ± 0.70 Bb
	THT	0.20 ± 0.41 Bb	9.20 ± 3.77 Bac	2.72 ± 1.70 Ab	0.04 ± 0.20 Aa	0.00 ± 0.00 Ba	0.28 ± 0.54 Aa	0.24 ± 0.52 Aa	3.44 ± 1.53 Aa	0.72 ± 1.02 Bb
Day 21	Control	0.68 ± 0.75 Aa	9.40 ± 4.70 Bac	2.00 ± 0.96 Aab	0.20 ± 0.41 Aa	0.00 ± 0.00 Ba	0.16 ± 0.37 Aa	0.36 ± 0.49 Aa	3.84 ± 2.29 Aa	1.08 ± 1.00 Aa
	HT	0.48 ± 0.65 Aab	11.20 ± 4.32 Aa	2.84 ± 2.15 Aa	0.24 ± 0.44 Aa	0.00 ± 0.00 Ba	0.16 ± 0.47 Aa	0.20 ± 0.41 Aa	3.44 ± 1.87 Aac	1.92 ± 1.47 Aa
	B	0.40 ± 0.58 Bab	6.56 ± 3.18 Bb	1.40 ± 1,41 Bbc	0.04 ± 0.20 Aa	0.00 ± 0.00 Ba	0.04 ± 0.20 Aa	0.04 ± 0.20 Ab	2.80 ± 1.32 Bab	0.16 ± 0.37 Bb
	BHT	0.16 ± 0.37 Bb	7.28 ± 3.29 Bc	1.24 ± 1.05 Bc	0.00 ± 0.00 Aa	0.00 ± 0.00 Ba	0.16 ± 0.37 Aa	0.04 ± 0.20 Ab	2.32 ± 2.17 Bb	0.28 ± 0.54 Bb
	T	0.32 ± 0.48 Bab	10.36 ± 3.39 Ab	1.88 ± 1.13 Aabc	0.24 ± 0.52 Aa	0.00 ± 0.00 Ba	0.32 ± 0.48 Aa	0.08 ± 0.28 Aa	2.36 ± 1.93 Bbc	0.40 ± 0.58 Bb
	THT	0.20 ± 0.50 Bb	5.84 ± 2.73 Bc	1.44 ± 1.16 Babc	0.08 ± 0.28 Aa	0.00 ± 0.00 Ba	0.20 ± 0.41 Aa	0.16 ± 0.37 Aa	2.52 ± 2.06 Bab	0.44 ± 0.65 Bb
Day 28	Control	0.92 ± 1.04 Aa	6.84 ± 2.79 Ba	3.24 ± 1.39 Aa	0.28 ± 0.46 Aa	0.08 ± 0.28 Aa	0.24 ± 0.52 Aa	0.28 ± 0.54 Aa	2.48 ± 1.48 Ba	0.76 ± 0.66 Bab
	HT	0.44 ± 0.58 Ba	12.68 ± 5.43 Ab	2.56 ± 1.61 Aa	0.04 ± 0.20 Aa	0.04 ± 0.20 Aa	0.12 ± 0.33 Aa	0.24 ± 0.44 Aa	3.08 ± 2.20 Ba	1.48 ± 1.48 Ab
	B	0.08 ± 0.28 Bb	5.00 ± 1.71 Bc	1.20 ± 1.08 Bb	0.04 ± 0.20 Aa	0.00 ± 0.00 Ba	0.00 ± 0.00 Aa	0.04 ± 0.20 Aa	0.48 ± 0.51 Bb	0.08 ± 0.28 Bc
	BHT	0.08 ± 0.28 Bb	3.44 ± 2.18 Bd	1.04 ± 0.89 Bb	0.00 ± 0.00 Ab	0.00 ± 0.00 Ba	0.08 ± 0.28 Aa	0.00 ± 0.00 Aa	0.24 ± 0.52 Bb	0.80 ± 1.22 Bab
	T	0.36 ± 0.57 Bab	6.32 ± 3.21 Bac	0.88 ± 0.73 Bb	0.12 ± 0.33 Aa	0.00 ± 0.00 Ba	0.04 ± 0.20 Aa	0.08 ± 0.28 Aa	1.20 ± 0.96 Bb	0.80 ± 0.91 Bab
	THT	0.36 ± 0.49 Ba	8.32 ± 4.98 Ba	1.44 ± 1.42 Bb	0.00 ± 0.00 Ab	0.00 ± 0.00 Ba	0.16 ± 0.37 Aa	0.12 ± 0.44 Aa	2.00 ± 1.35 Ba	0.52 ± 0.65 Ba

**Table 5:** Average number (and standard deviation) of flagellate protists belonging to different morphotypes (n1 to n9) after 1, 7, 14, 21 and 28 days of exposure; termites were exposed to untreated pine (Pine), untreated beech (Control), heat-treated beech (HT), beech treated with: Bicine (B), Bicine and heat treatment (BHT), Tricine (T), Tricine and heat treatment (THT). Different capital letters within the same column indicate significant differences (*p* < 0.05) between pine and the rest of the treatments for each day of observation; different small letters within the same column indicate significant differences (*p* < 0.05) among spruce treatments for each day of observation.

	Treatment	n1	n2	n3	n4	n5	n6	n7	n8	n9
	Pine	0.86 ± 0.78 A	12.06 ± 3.28 A	2.62 ± 1.84 A	0.24 ± 0.48 A	0.30 ± 0.65 A	0.06 ± 0.24 A	0.22 ± 0.58 A	4.56 ± 2.84 A	1.42 ± 1.05 A
Day 1	Control	0.52 ± 0.65 Aa	9.32 ± 2.91 Ba	4.08 ± 2.33 Ba	0.24 ± 0.44 Aa	0.12 ± 0.33 Aa	0.12 ± 0.33 Aa	0.16 ± 0.37 Aa	2.44 ± 1.73 Ba	1.36 ± 1.04 Aa
	HT	0.72 ± 0.68 Aa	8.88 ± 2.91 Bab	3.04 ± 1.43 Aa	0.20 ± 0.50 Aa	0.28 ± 0.46 Aa	0.12 ± 0.33 Aa	0.28 ± 0.46 Aa	3.28 ± 2.21 Aa	1.44 ± 1.26 Aa
	B	0.64 ± 0.70 Aa	7.20 ± 3.37 Bab	3.12 ± 1.81 Aa	0.20 ± 0.41 Aa	0.16 ± 0.47 Aa	0.12 ± 0.33 Aa	0.16 ± 0.37 Aa	2.52 ± 2.33 Ba	0.64 ± 0.86 Bb
	BHT	0.64 ± 0.86 Aa	7.32 ± 4.41 Bb	2.80 ± 1.66 Aab	0.32 ± 0.56 Aa	0.24 ± 0.44 Aa	0.16 ± 0.37 Aa	0.08 ± 0.28 Aa	2.12 ± 1.42 Ba	0.72 ± 0.79 Bab
	T	0.40 ± 0.50 Aa	7.48 ± 2.87 Bab	3.48 ± 1.83 Aa	0.08 ± 0.28 Aa	0.08 ± 0.28 Aa	0.04 ± 0.20 Aa	0.16 ± 0.37 Aa	1.96 ± 1,37 Ba	0.88 ± 0.88 Aab
	THT	0.48 ± 0.65 Aa	4.72 ± 1.40 Bc	1.76 ± 1.16 Ab	0.04 ± 0.20 Aa	0.04 ± 0.20 Aa	0.04 ± 0.20 Aa	0.08 ± 0.28 Aa	1.96 ± 1,49 Ba	1.28 ± 0.89 Aa
Day 7	Control	0.72 ± 0.84 Aa	10.32 ± 3.41 Ba	2.80 ± 1.71 Aa	0.16 ± 0.37 Aa	0.00 ± 0.00 Aa	0.12 ± 0.33 Aa	0.32 ± 0.56 Aa	3.72 ± 3.70 Aa	1.28 ± 0.98 Aac
	HT	0.48 ± 0.51 Aa	10.2 ± 3.50 Bac	2.48 ± 1.56 Aab	0.32 ± 0.63 Aa	0.00 ± 0.00 Aa	0.04 ± 0.20 Aa	0.16 ± 0.47 Aa	2.76 ± 1.20 Aa	1.20 ± 0.87 Aac
	B	0.64 ± 0.70 Aa	8.28 ± 2.64 Bb	1.60 ± 1.26 Abc	0.16 ± 0.37 Aa	0.00 ± 0.00 Aa	0.12 ± 0.33 Aa	0.20 ± 0.41 Aa	3.60 ± 3.23 Aa	0.84 ± 0.75 Bb
	BHT	0.64 ± 0.76 Aa	9.48 ± 4.45 Bbc	2.52 ± 1.73 Aa	0.12 ± 0.44 Aa	0.08 ± 0.28 Aa	0.20 ± 0.50 Aa	0.20 ± 0.50 Aa	3.16 ± 1.97 Aa	1.16 ± 0.99 Bab
	T	0.36 ± 0.57 Bab	10.6 ± 5.02 Bab	3.04 ± 1.81 Aa	0.08 ± 0.28 Aa	0.04 ± 0.20 Aa	0.08 ± 0.28 Aa	0.24 ± 0.44 Aa	6.28 ± 2.70 Ba	0.88 ± 0.73 Bbc
	THT	0.16 ± 0.47 Bb	6.96 ± 2.37 Bc	1.52 ± 0.96 Bc	0.08 ± 0.28 Aa	0.00 ± 0.00 Ba	0.16 ± 0.37 Aa	0.12 ± 0.33 Aa	4.60 ± 2.50 Ba	0.36 ± 0.70 Bb
Day 14	Control	0.72 ± 0.74 Aa	12.40 ± 3.52 Aa	3.44 ± 1.87 Aa	0.24 ± 0.44 Aa	0.00 ± 0.00 Ba	0.28 ± 0.46 Aa	0.20 ± 0.50 Aa	3.84 ± 2.15 Aa	1.88 ± 1.30 Aa
	HT	0.60 ± 0.71 Aab	8.64 ± 3.38 Bbc	2.84 ± 1.68 Aa	0.16 ± 0.37 Aa	0.04 ± 0.20 Aa	0.08 ± 0.28 Aa	0.16 ± 0.37 Aa	4.56 ± 2.57 Aa	1.56 ± 1.33 Aa
	B	0.32 ± 0.48 Bab	8.40 ± 3.55 Bbc	2.36 ± 1.55 Aa	0.12 ± 0.33 Aa	0.00 ± 0.00 Ba	0.20 ± 0.50 Aa	0.12 ± 0.33 Aa	3.56 ± 2.72 Aa	0.40 ± 0.58 Bb
	BHT	0.28 ± 0.54 Bab	12.4 ± 3.99 Aa	3.64 ± 2.27 Aa	0.20 ± 0.41 Aa	0.00 ± 0.00 Ba	0.08 ± 0.28 Aa	0.24 ± 0.44 Aa	3.96 ± 2.84 Aa	0.72 ± 0.68 Bb
	T	0.52 ± 0.71 Aab	9.16 ± 2.84 Bb	2.32 ± 1.49 Aa	0.12 ± 0.33 Aa	0.00 ± 0.00 Ba	0.24 ± 0.52 Aa	0.24 ± 0.52 Aa	3.96 ± 2.79 Aa	0.64 ± 0.76 Bb
	THT	0.20 ± 0.41 Bb	7.24 ± 2.59 Bc	2.92 ± 2.06 Aa	0.08 ± 0.28 Aa	0.00 ± 0.00 Ba	0.12 ± 0.33 Aa	0.28 ± 0.61 Aa	4.32 ± 2.85 Aa	0.84 ± 0.90 Ba
Day 21	Control	0.64 ± 0.70 Aa	9.80 ± 3.32 Ba	3.48 ± 1.83 Ba	0.24 ± 0.52 Aa	0.00 ± 0.00 Ba	0.20 ± 0.41 Aa	0.20 ± 0.41 Aa	3.00 ± 1.76 Bab	1.40 ± 1.00 Aa
	HT	0.48 ± 0.59 Aa	8.84 ± 3.30 Bab	3.68 ± 2.17 Bab	0.12 ± 0.33 Aa	0.04 ± 0.20 Aa	0.12 ± 0.33 Aa	0.24 ± 0.44 Aa	3.56 ± 2.26 Bb	1.32 ± 1.38 Aa
	B	0.20 ± 0.41 Ba	10.28 ± 4.77 Ba	1.48 ± 0.87 Abc	0.04 ± 0.20 Aa	0.00 ± 0.00 Ba	0.24 ± 0.52 Aa	0.12 ± 0.33 Aa	2.08 ± 1.35 Ba	0.48 ± 0.71 Bb
	BHT	0.36 ± 0.64 Aa	8.60 ± 2.92 Bb	1.76 ± 1.16 Abc	0.08 ± 0.28 Aa	0.00 ± 0.00 Ba	0.08 ± 0.28 Aa	0.04 ± 0.20 Aa	1.84 ± 1.72 Ba	0.84 ± 1.52 Bb
	T	0.48 ± 0.65 Aa	8.56 ± 3.81 Bb	2.16 ± 1.97 Ab	0.12 ± 0.33 Aa	0.00 ± 0.00 Ba	0.08 ± 0.28 Aa	0.12 ± 0.33 Aa	2.04 ± 2.41 Ba	1.36 ± 1.82 Aab
	THT	0.16 ± 0.37 Ba	7.80 ± 2.00 Bc	1.72 ± 1.49 Bc	0.16 ± 0.37 Aa	0.00 ± 0.00 Ba	0.12 ± 0.33 Aa	0.04 ± 0.20 Aa	3.00 ± 2.31 Bab	0.80 ± 1.12 Aab
Day 28	Control	0.76 ± 0.72 Aa	8.72 ± 3.47 Bab	2.52 ± 1.42 Aa	0.40 ± 0.87 Aa	0.00 ± 0.00 Ba	0.36 ± 1.04 Aa	0.16 ± 0.37 Aa	2.12 ± 1.13 Ba	0.92 ± 0.91 Ba
	HT	0.28 ± 0.46 Ba	9.28 ± 2.69 Bb	2.84 ± 1.86 Aa	0.04 ± 0.20 Aab	0.00 ± 0.00 Ba	0.16 ± 0.37 Aa	0.08 ± 0.28 Aa	1.04 ± 0.93 Bbd	1.16 ± 0.94 Aa
	B	0.12 ± 0.33 Ba	7.32 ± 4.49 Bac	1.32 ± 1.25 Bb	0.00 ± 0.00 Ab	0.00 ± 0.00 Ba	0.20 ± 0.50 Aa	0.24 ± 0.44 Aa	1.12 ± 1.09 Bbd	0.16 ± 0.47 Bb
	BHT	0.24 ± 0.44 Ba	5.40 ± 2.52 Bc	1.76 ± 1.64 Ab	0.20 ± 0.41 Aab	0.00 ± 0.00 Ba	0.08 ± 0.28 Aa	0.08 ± 0.28 Aa	0.88 ± 0.97 Bbc	0.08 ± 0.40 Bb
	T	0.52 ± 0.51 Aa	5.84 ± 2.27 Bc	1.48 ± 1.16 Bb	0.00 ± 0.00 Ab	0.00 ± 0.00 Ba	0.04 ± 0.20 Aa	0.12 ± 0.44 Aa	0.40 ± 0.71 Bc	0.16 ± 0.37 Bb
	THT	0.08 ± 0.28 Ba	6.80 ± 2.25 Bac	1.40 ± 1.08 Bb	0.04 ± 0.20 Aab	0.00 ± 0.00 Ba	0.12 ± 0.33 Aa	0.08 ± 0.28 Aa	1.68 ± 1.25 Bad	0.08 ± 0.28 Bb

## Data Availability

Not applicable.
